# A Dual-Channel and Multi-Sensor Fusion Framework for Coal Mine Image Dehazing

**DOI:** 10.3390/s26103171

**Published:** 2026-05-17

**Authors:** Xinliang Wang, Yan Huo

**Affiliations:** School of Intelligent Science and Information Engineering, Shenyang University, Shenyang 110044, China

**Keywords:** image dehazing, adaptive bright–dark channel fusion, multi-sensor complementary, weighted ambient light estimation, weighted guided filter

## Abstract

Due to dust, haze and uneven lighting conditions, images captured in coal mines frequently suffer severe quality degradation. Traditional dehazing methods typically overlook color characteristics and employ single algorithms, and deep-learning-based approaches require substantial training data and demand high hardware specifications, which restricts their dehazing performance and efficiency. This research proposes an efficient image dehazing framework. This method integrates bright and dark channel information to derive contrast feature values based on their linear differences. These values reflect dust concentration levels in the environment. By incorporating dust sensor data, the adaptive scaling coefficient and dust compensation terms are established. The adaptive scaling coefficient serves as a dynamic pixel selection ratio during ambient light estimation, effectively preserving the brightest pixel points. The global color mean functions as the criterion for determining image color characteristics, distinguishing between color images and low-light grayscale images to enable different dehazing approaches. This process achieves state verification and information complementarity between visual perception and dust measurement. The weighted fusion of bright and dark channels yields more accurate estimation for ambient light and transmission. Additionally, a weighted guided filter is designed with dust compensation terms incorporated. Ablation studies were conducted to validate the effectiveness of this method in enhancing image features. Finally, comparative experiments were performed using a self-constructed coal mine hazy image dataset, along with SOTS-indoor and SOTS-outdoor datasets. Experimental results demonstrate that, compared with other state-of-the-art methods, this method effectively removes haze while restoring image features and details, exhibiting superior stability, adaptability, and computational efficiency.

## 1. Introduction

Computer vision technology is increasingly vital for intelligent mining operations. The underground coal mine environment presents unique difficulties for image acquisition and processing. The limited underground space depends mainly on artificial lighting. This creates uneven lighting conditions across the area. Dust particles and water vapor from mining activities scatter and absorb light. These suspended particles create a haze effect in captured images. The haze causes blurring and reduces image contrast significantly. Image details are lost as a result. This limits the effectiveness of computer vision applications in mines [1,2,3]. Research on haze removal for coal mine images is therefore necessary. Better image quality supports key mining applications. These applications include safety monitoring, worker positioning, and equipment fault identification. This research has practical importance for worker safety and intelligent mining development.

Some scholars have studied image degradation and haze removal extensively. Current methods fall into three main categories. They are traditional image enhancement techniques, deep-learning-based methods, and physical-model-based approaches. Each category has distinct characteristics:

(1) Traditional image enhancement methods emerged early in this field, including histogram equalization [4] and homomorphic filtering [5]. These techniques improve contrast by modifying pixel grayscale distribution. Global processing often ignores local features, which can cause loss of fine details. Local over-enhancement may also occur.
(2) Deep-learning-based methods have advanced rapidly in recent years. These approaches use deep learning frameworks. They learn the mapping from hazy to clear images. Model training requires large paired datasets of hazy and haze-free images. Notable methods include DehazeNet [6], AOD-Net [7], PSD [8], RIDCP [9], and MaIR [10]. These methods show good results on natural image datasets, whereas they face challenges in complex real environments. Underwater and coal mining scenarios present difficulties. These models have weaker interpretability than physical model approaches. Obtaining large real-world paired datasets remains difficult. Models trained on synthetic data generalize poorly to real applications. High computational requirements limit deployment on embedded devices [11,12,13].
(3) Physical-model-based methods can be divided into two main categories. The first category uses Retinex theory and its derivatives [14,15]. These methods separate images into illumination and reflectance components. They perform well in color fidelity and detail enhancement, but parameter tuning presents challenges. Computational complexity is often high. Adaptability to complex scenes remains limited. The second category uses the atmospheric scattering model [16,17]. These methods recover clear images by solving the model. Researchers have developed various prior knowledge approaches. He et al. [18] introduced the dark channel prior (DCP). Wang et al. [19] proposed the bright channel prior (BCP). These methods produce natural results with high fidelity. However, using only the DCP can cause color distortion and darkening, and relying solely on the BCP may lose shadow details. Combining the BCP and DCP offers complementary benefits. This approach shows promise for coal mine image dehazing.

In response to the challenges of limited real-world coal mine hazy images and practical requirements for lightweight terminal deployment, this study proposes an image dehazing framework based on bright–dark channel and multi-sensor fusion.

Our main contributions are as follows:

- We propose a criterion based on the global color mean to distinguish image color features. This criterion effectively separates color images from low-light grayscale images. Different processing strategies are applied based on this criterion. The approach improves overall dehazing quality.
- We design a complementary strategy using visual sensing and dust concentration measurement. A dust measurement compensation module is added to the visual signal processing pipeline. This enables complementary use and cross-validation of heterogeneous data. The approach automatically detects abnormal sensor states and improves the environmental adaptability of different sensors.
- We propose an adaptive fusion method for the BCP and DCP. Linear computation of these priors yields contrast features and adaptive scaling coefficients. A dynamic brightest pixel selection mechanism and weighted constraint terms are established. These components enable fusion of bright and dark channel features. The method automatically adjusts parameters based on different scenes. This improves accuracy in ambient light and transmission estimation.
- We design a weighted guided filter via the adaptive regularization weight and dual-window strategy. This approach optimizes image features while preserving edge details.

This paper is organized as follows: Section 2 introduces related works. Section 3 describes the proposed method in detail. Section 4 presents the experiments and results. The conclusion is given in Section 5.

## 2. Related Works

Physical-model-based image dehazing methods demonstrate effectiveness through their strong model interpretability. The atmospheric scattering model represents a classical framework in this domain. Its dehazing performance has improved significantly through the integration of prior knowledge. He et al. [18] made a landmark contribution with the DCP. This prior relies on statistical observations of haze-free outdoor images. It proposes that most local regions (excluding sky areas) contain at least one-color channel with very low intensity values. This characteristic enables estimation of transmission maps and atmospheric light. Researchers have developed improvements to address the DCP’s limitations in bright regions. Wang et al. [19] introduced the BCP, which states that local areas of haze-free images show at least one-color channel with relatively high pixel values. This prior helps refine atmospheric light estimation.

Recent DCP and BCP research shows several clear trends. Application scenarios have expanded from outdoor haze processing to complex environments like underwater and mining settings. Prior theories themselves have undergone improvement through adaptive parameter tuning and model optimization. Integration with deep learning has emerged, utilizing fitting capabilities of neural networks to learn complex prior information and optimize key parameters. Multiple prior knowledge frameworks now merge, combining the BCP and DCP with other theories. This fusion compensates for individual prior limitations and enhances overall dehazing performance.

For prior theories, He et al. [20] improved transmission estimation using the DCP and guided filtering. This approach preserves edge information while reducing halo artifacts. Drews Jr et al. [21] calculated dark channels using only green and blue channels for underwater environments. This method increased transmission estimation accuracy. Bao et al. [22] developed adaptive parameter adjustment for mixed haze and low-light conditions. The mechanism adjusts transmission parameters based on image brightness and contrast. It addresses underestimation problems in low-light areas. Sun et al. [23] applied the BCP to detect high-intensity pixels in color channels. They combined this with grayscale removal to improve image display quality. Single-prior theories work well for color-rich images. They still face challenges in complex scenarios like mining and remote sensing.

For combining prior theories with deep learning, Tao et al. [24] combined a CNN with the BCP for image enhancement. The CNN performs denoising while the BCP estimates transmission maps. This integration significantly improves image features. Lee et al. [25] used the BCP as a physical constraint with unsupervised adversarial learning. Their method optimizes brightness distribution from low-light to normal illumination. Yang et al. [26] proposed MSDN-DCP with feature extraction and fusion modules. The dark channel refinement module enhances feature maps. This approach improves image evaluation metrics. Han et al. [27] designed a lightweight U-net architecture based on the DCP. It maintains dehazing performance while reducing parameters and computational complexity. Deep learning methods show strong performance but have limited interpretability. Their effectiveness depends heavily on large-scale datasets.

On the fusion of different prior theories, Jiang et al. [28] developed an adaptive dual-channel prior method. This approach dynamically selects between the DCP and BCP based on image global characteristics. The selection improves algorithm robustness across different scenarios. Fan et al. [29] implemented bright–dark channel segmentation and fusion. The dark channel removes dust haze and enhances details. The bright channel estimates illumination and corrects colors. This strategy preserves image details while maintaining natural visual appearance. Cao et al. [30] focused on underground coal mine environments. They combined multi-scale guided filtering with adaptive atmospheric light estimation. This method addresses effects of multi-scale dust particles. It achieves precise segmentation of image regions. The approach significantly enhances atmospheric light estimation accuracy. Fusion strategies provide viable solutions for complex scenario dehazing. Environmental adaptability needs further validation across diverse conditions.

## 3. Proposed Method

### 3.1. Overall Architecture

**Figure 1:**
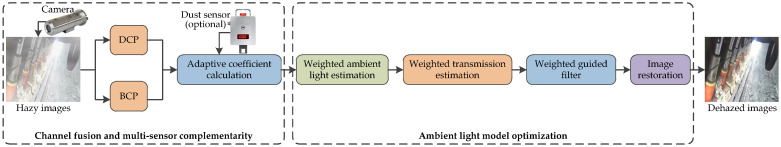
The architecture of our proposed adaptive bright–dark channel fusion (ABDCF).

This study presents a coal mine image dehazing framework using multi-sensor complementarity and bright–dark channel fusion. As shown in Figure 1, the method processes input hazy images through several steps. The BCP and DCP are applied separately. Linear difference calculation between dual-channel features yields contrast features. An adaptive scaling coefficient is derived from these features. This coefficient determines the dynamic selection ratio for the brightest pixels in ambient light estimation. The dust sensor functions as an optional component and, when incorporated into our framework, dust concentration values serve as compensation terms in ambient light estimation. Cross-validation analysis of contrast features and dust concentration enables automatic sensor anomaly detection. The global color mean of images provides the basis for color feature discrimination. Different parameter adjustments are applied to color images and low-light grayscale images. A weighting strategy achieves effective fusion of bright and dark channels. This fusion enables weighted ambient light estimation and weighted transmission estimation. The weighted guided filter is designed to enhance image edge details. Image restoration uses the degradation model for hazy images. The framework involves two main processes: channel fusion with multi-sensor complementarity and ambient light model optimization.

### 3.2. Channel Fusion and Multi-Sensor Complementarity

The DCP builds on a key observation about haze-free images. Most local regions, excluding sky areas, contain pixels with very low intensity in at least one-color channel. For any natural image *J*, its dark channel can be expressed as:

(1) $$J^{dark}(x)=\underset{c\in \left\{R,G,B\right\}}{\min}(\underset{y\in \Omega (x)}{\min}J^{c}(y)),$$

where *x* represents the current pixel location, *y* indicates pixel positions within the neighborhood, and *c* denotes the color channel (R, G, or B). Ω(*x*) is the neighborhood window centered at pixel *x*, and $J^{c}(y)$ represents the pixel value of image *J* in a specific color channel. According to the DCP theory, the dark channel intensity of haze-free images is very low in local non-sky regions, approaching zero.

The fundamental principle of the BCP is that, in hazy images, certain pixels exhibit high intensity in specific channels. Based on the BCP theory, the bright channel intensity at a given pixel *x* approximates the atmospheric light intensity of the haze-free image. For any color image *J*, its bright channel can be expressed as:

(2) $$J^{bright}(x)=\underset{c\in \left\{R,G,B\right\}}{\max}(\underset{y\in \Omega (x)}{\max}J^{c}(y)),$$

In the complex environment of coal mines, the BCP and DCP exhibit distinct characteristics when processing images of different color types. Applying the BCP to color images tends to introduce distortion, yet it effectively enhances texture details in grayscale images. Conversely, using the DCP on grayscale images often overlooks local features, but it improves detail preservation in color images. Therefore, the color characteristics of an image can serve as a critical basis for channel fusion.

This study employs the global color mean to identify the color features of an image. The input image can be represented as $I=R^{H\times W\times 3}$, where *H* and *W* denote the image height and width, respectively, and 3 corresponds to the RGB color channels. The image is flattened into a one-dimensional sequence with total pixel count *N* = *H* × *W*. For the pixel i (i∈{1,2,…,N}), its RGB channel values are expressed as $I_{c[i]}$ (c∈{R,G,B}). The local maximum $I_{max[i]}$ and local minimum $I_{min[i]}$ are defined as:

(3) $$\begin{array}{l} I_{\max[i]}=\max_{c}\left(I_{c[i]}\right)\\ I_{\min[i]}=\min_{c}\left(I_{c[i]}\right) \end{array},$$

where $I_{max[i]}$ and $I_{min[i]}$ represent the local extreme values across the three channels for a single pixel i. The local color ratio $r_{i}$ is calculated as:

(4) $$r_{i}=\frac{I_{\max[i]}}{I_{\min[i]}},$$

**Figure 2:**
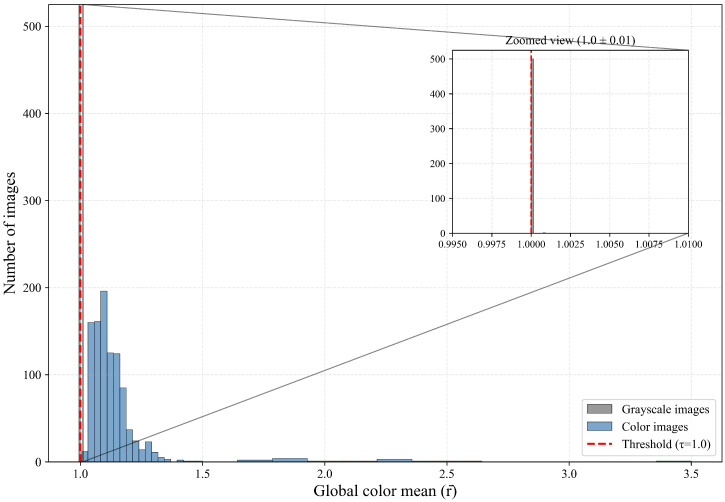
Statistical analysis results of images with different color characteristics.

In color images, the two extreme values differ significantly, resulting in $r_{i}>1$. Conversely, for grayscale images, the extremes tend to be similar. To characterize the overall color properties of the image, the global color mean is computed as:

(5) $$\bar{r}=\frac{1}{N}\sum_{i=1}^{N}r_{i},(\tau =1),$$

where *τ* represents the determination threshold, *τ* = 1, when $\bar{r}>1$, this indicates the presence of at least one colored pixel in the image, whereas $\bar{r}\leq 1$ suggests a grayscale image. To verify the effectiveness of global color mean in distinguishing image color characteristics, we conducted statistical analysis using a self-constructed coal mine hazy image dataset. This dataset comprises 1500 images, including 1000 color images and 500 low-light grayscale images. The statistical results are presented in Figure 2, the global color mean values range from 1 to 3.5. All low-light grayscale images exhibit a global color mean of exactly 1. For color images, most global color mean values fall within the interval (1, 1.5], while images in other ranges are relatively scarce. Therefore, the global color mean criterion and the selection of threshold *τ* = 1 are justified.

When an image is input, the dark channel value $J^{dark}(x)$ is obtained using Equation (1). For notational simplicity, let $D\left(x\right)=J^{dark}(x)$. The image is then processed using the BCP, yielding the bright channel value $J^{bright}(x)$ according to Equation c. Similarly, let $B\left(x\right)=J^{bright}(x)$ for brevity. To ensure consistent processing across images with different bit depths, if the image data type is unsigned 8-bit integer (uint8), where pixel values range from [0, 255], the values are normalized to the [0, 1] interval by dividing by 255. Otherwise, the original values are retained. The resulting normalized dark and bright channel values are denoted as *D*_n_(*x*) and *B*_n_(*x*), respectively, as expressed in the following equations:

(6) $$\begin{array}{l} D_{n}(x)=\left\{\begin{array}{c} \frac{D(x)}{255},\;\mathrm{if}\;\mathrm{dtype}=\mathrm{uint}8\\ \;\;D(x),\;\mathrm{otherwise} \end{array}\right.\\ B_{n}(x)=\left\{\begin{array}{c} \frac{B(x)}{255},\;\mathrm{if}\;\mathrm{dtype}=\mathrm{uint}8\\ \;\;B(x),\;\mathrm{otherwise} \end{array}\right. \end{array},$$

Subsequently, the mean values of the dark channel ($\mu_{dark}$) and the bright channel ($\mu_{bright}$) for the entire image are calculated using the following equations:

(7) $$\begin{array}{l} \mu_{dark}=\frac{1}{N}\sum D_{n}(x)\\ \mu_{bright}=\frac{1}{N}\sum B_{n}(x) \end{array},$$

where *N* represents the total number of pixels in the image and *x* denotes each pixel position to be traversed.

**Figure 3:**
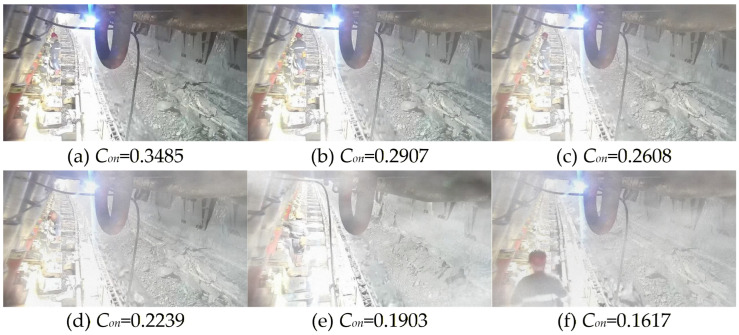
The relationship between the value of *C_on_* and dust concentration.

In the subsequent ambient light estimation step, a certain proportion of the brightest pixels needs to be selected. Instead of using a fixed value, a more reasonable adaptive scaling coefficient, *Coef*, is adopted. The calculation of this coefficient begins with deriving the contrast feature value *C_on_* through a linear difference based on the global image features $\mu_{dark}$ and $\mu_{bright}$. The calculation equations for *C_on_* and *Coef* are as follows:

(8) $$\begin{array}{l} C_{on}=\mu_{bright}-\mu_{dark}\\ Coef=\frac{C_{on}+\eta }{R}\\ \eta =\frac{D_{v}}{1000} \end{array},$$

where *R* denotes the proportional adjustment parameter. Depending on the color characteristics of images, *R* ranges from [1, 30] for color images and [40, 80] for low-light grayscale images. *η* represents the dust measurement compensation term, which varies linearly with the dust concentration value and ranges from [0, 1]. *D_v_* denotes the actual measured value of dust concentration. The GCG1000 dust sensor was selected for dust measurement, with a measurement range of 0–1000 mg/m^3^. Dust concentration values are transmitted through underground monitoring substations and underground network switches to the ground monitoring center. Video signals are transmitted via local video transmission switches and underground network switches to the ground monitoring center. Although dust data transmission and video signal transmission follow separate paths, the backbone network employs fiber optic transmission, ensuring real-time synchronous data acquisition. As illustrated in Figure 3, the contrast feature value *C_on_* can be used to characterize the dust concentration level in the environment. The value of *C_on_* is inversely proportional to dust concentration.

**Figure 4:**
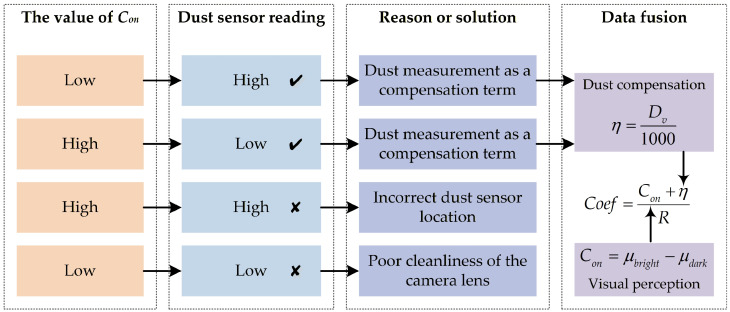
Heterogeneous data fusion verification of camera and dust sensor.

The complementary validation process between visual perception and dust measurement is illustrated in Figure 4. When the camera lens is clean, the captured images accurately reflect the actual environmental conditions. Under normal circumstances, the dust sensor measurements should exhibit a consistent correlation with the *C_on_* value: a lower *C_on_* value corresponds to higher sensor readings, while a higher *C_on_* value corresponds to lower sensor readings. In such cases, the transformed dust measurement value can be incorporated as the compensation term *η* in subsequent calculations. However, when discrepancies arise between the visual characteristics of the image and the dust sensor readings, the system is in an abnormal state. Two primary abnormal scenarios are identified:

(1) Low image clarity coupled with low dust sensor readings, which typically indicates contamination of the camera lens.
(2) Relatively clear images accompanied by high dust sensor readings, which may result from improper installation of the dust sensor.

By integrating heterogeneous data from both the camera and the dust sensor, these anomalies can be effectively identified and mitigated, thereby ensuring the accuracy of ambient light estimation. Finally, Equation (8) is employed to accomplish the data fusion between the dust compensation term *η* and the visual perception term *C_on_*.

### 3.3. Ambient Light Model Optimization

In the classical atmospheric light scattering model described as *I*(*x*) = *J*(*x*)*t*(*x*) + *A* [1 − *t*(*x*)], the term *J*(*x*)*t*(*x*) is the direct attenuation component, which describes the attenuation of reflected light *J*(*x*) as it passes through different media, and this component decreases as the propagation distance increases or the medium concentration rises. *A* represents the atmospheric light value. However, in underground coal mine environments, illumination is typically insufficient and unevenly distributed, precluding the existence of a globally uniform atmospheric light value *A*. Consequently, the atmospheric light scattering model can be reformulated as follows:

(9) I(x)=J(x)t(x)+L(x)[1−t(x)],

where *I*(*x*) represents the actual hazy image, while *L*(*x*) denotes the ambient light energy at pixel location *x*. The term *L*(*x*)[1 − *t*(*x*)] is the ambient light component, representing the portion of ambient light *L*(*x*) scattered by dust particles into the image sensor. This component is the primary cause of reduced image contrast and color distortion. Additionally, considering that excessively small values of transmission *t*(*x*) may introduce noise, it is necessary to impose constraints on the range of *t*(*x*). By modifying Equation (9), the degradation model for hazy images in coal mines can be expressed as follows:

(10) $$J(x)=\frac{I(x)-L(x)}{\max(t_{0},t(x))}+L(x),$$

where *t*_0_ denotes the minimum transmission, which is set to 0.1 in this study. Image dehazing and restoration are based on this equation. Given a hazy input image, *I*(*x*), to obtain the clear image *J*(*x*), it is necessary to first estimate the ambient light value *L*(*x*) and the transmission *t*(*x*).

The estimation of the ambient light value *L*(*x*) is complicated in underground coal mines due to the presence of multiple artificial light sources. Direct and reflected light from these sources often create localized high-brightness regions, which can lead to inaccurate ambient light estimation and subsequent image distortion. To address this issue, this study employs a weighted dual-channel fusion averaging method. The implementation process is as follows:

First, the total number of pixels *N* in the input hazy image is calculated. Based on the results of dual-channel processing, an adaptive scaling coefficient *Coef* (i.e., the pixel selection ratio) is determined to obtain the number of candidate pixels *N*_s_ (*N*_s_ = *Coef* × *N*). By integrating the dark channel value *D*(*x*) and the bright channel value *B*(*x*), a joint feature value $W_{c}$ is established. The indices corresponding to the *N*_s_ pixels with the largest $W_{c}$ values are selected. Using these indices, the corresponding pixels are retrieved, and the average of their RGB values is computed as the ambient light value. The equation for calculating the joint feature value $W_{c}$ is as follows:

(11) $$W_{c}=\alpha B\left(x\right)+(1-\alpha )D\left(x\right),$$

where *α* represents the weighting coefficient, which ranges between 0 and 1. In this study, *α* is set to 0.8. This approach ensures that brighter regions in both the dark channel and the bright channel are considered, thereby reducing reliance on a single channel and enabling the extraction of more comprehensive image features.

For the estimation of transmission *t*(*x*), the hazy image *I*(*x*) is first normalized relative to the ambient light value *L*(*x*) to eliminate the influence of ambient light intensity on the calculation. The normalized result, denoted as $I_{n}(x)$, is expressed as follows:

(12) $$I_{n}(x)=\frac{I(x)}{L(x)},$$

Based on the calculation equation for the dark channel and bright channel, the dark channel $J^{dark}(x)$ and bright channel $J^{bright}(x)$ of the normalized result $I_{n}(x)$ are computed respectively:

(13) $$\begin{array}{l} J^{dark}(x)=\underset{c\in \left\{R,G,B\right\}}{\min}(\underset{y\in \Omega (x)}{\min}I_{n}^{c}(y))\\ J^{bright}(x)=\underset{c\in \left\{R,G,B\right\}}{\max}(\underset{y\in \Omega (x)}{\max}I_{n}^{c}(y)) \end{array},$$

To integrate the characteristics of both the dark channel and the bright channel, the contrast feature value *C_on_* is utilized to derive the weighting coefficient *β* through linear transformation, where *C_on_* is an integer multiple of *β*. By combining Equations (12) and (13), the estimation equation for transmission *t*(*x*) is obtained as follows:

(14) $$t(x)=1-(1-\beta )\underset{c\in \left\{R,G,B\right\}}{\min}\left(\underset{y\in \Omega (x)}{\min}\frac{I^{c}(y)}{L^{c}(x)}\right)+\beta \underset{c\in \left\{R,G,B\right\}}{\max}\left(\underset{y\in \Omega (x)}{\max}\frac{I^{c}(y)}{L^{c}(x)}\right),$$

The transmission map obtained through the above estimation process often exhibits halo artifacts. To address this issue, He et al. [20] proposed the soft-matting method and the guided filtering algorithm for refining the transmission map. The soft-matting method yields favorable results, but it is computationally intensive. Meanwhile, the guided filtering algorithm often fails to fully remove haze and tends to lose edge information.

In this paper, an adaptive regularization weight is introduced, and a weighted guided filtering algorithm is designed. The weighted guided filtering method requires two types of images: a guidance image and an estimation image. The guidance image is obtained by converting the input image to grayscale and applying normalization processing. The estimation image represents the transmission estimation *t*(*x*) derived from Equation (14). To simplify the algorithm description, the guidance image is denoted as *I*, and the estimation image is represented as *p*. The workflow of this algorithm is outlined in Algorithm 1, and a dual-window strategy is adopted. First, the mean filtering (with a 3 × 3 window) is applied for small-window convolution. When the total number of pixels in the guidance image is *N*, the local mean filtering value ${mean}_{I}$, the mean filtering result ${corr1}_{I}$ of the squared guidance image, and the local variance ${var1}_{I}$ of the guidance image are calculated. Using the normalized statistical value *S* and the weight adjustment parameter *δ*, an adaptive regularization weight *W* is constructed. Next, a large-window mean filtering operation with radius *r* is performed to compute local features of the guidance image *I* and the estimation image *p*. These include the local mean filtering value ${mean}_{I}$ of the guidance image, the local mean filtering result ${mean}_{p}$ of the estimation image, the mean filtering result ${corr2}_{I}$ of the squared guidance image, the mean filtering result ${corr}_{Ip}$ of the product of the guidance image and the estimation image, the local variance ${var2}_{I}$ of the guidance image, and the covariance ${cov}_{Ip}$ between the guidance image and the estimation image. During the calculation of the regularization parameter *ε* and the linear coefficients *a* and *b*, the adaptive regularization weight *W* is incorporated. By adjusting the weight *W*, the regularization strength *ε*/*W* can be reduced in texture-rich regions to preserve edge details, while it can be enhanced in texture-deficient regions to promote smoothing. Subsequently, mean filtering is applied to the linear coefficients *a* and *b* to obtain ${mean}_{a}$ and ${mean}_{b}$, respectively. Finally, the output image *q* is derived through linear transformation.
**Algorithm 1:** Weighted guided filter.**Input:** guidance image *I*, estimation image *p*, radius *r*, regularization *ε,* small value *δ* (prevents zero denominator).**Output:** filtering output *q*.               $\begin{array}{l} mean_{I}=f_{mean}(I)\\ corr1_{I}=f_{mean}(I.*I)\\ \mathrm{var}1_{I}=corr1_{I}-mean_{I^{.}}*mean_{I}\\ S=\sum_{i=1}^{N}(1/(\mathrm{var}1_{I}(i)+\delta ))/N\\ W=(\mathrm{var}1_{I}+\delta )\cdot S\\ mean_{I}=f_{mean}(I)\\ mean_{p}=f_{mean}(p)\\ corr2_{I}=f_{mean}(I.*I)\\ corr_{Ip}=f_{mean}(I.*p)\\ \mathrm{var}2_{I}=corr2_{I}-mean_{I^{.}}*mean_{I}\\ {\mathrm{cov}}_{Ip}=corr_{Ip}-mean_{I^{.}}*mean_{p}\\ a={\mathrm{cov}}_{Ip}./\left(\mathrm{var}2_{I}+\varepsilon /W\right)\\ b=mean_{p}-a.*mean_{I}\\ mean_{a}=f_{mean}(a)\\ mean_{b}=f_{mean}(b)\\ q=mean_{a}.*I+mean_{b} \end{array}$/*$f_{mean}$ is a mean filter with a wide variety of O(*N*) time methods. */

By employing the weighted guided filtering method, the transmission map can be optimized. This approach not only ensures effective haze removal but also enhances image edges. Substituting the estimated ambient light *L*(*x*) and the optimized transmission *t*(*x*) into Equation (10) yields the dehazed image *J*(*x*).

## 4. Experiments and Results

In this section, we sequentially detail the establishment of the experimental environment, image acquisition and preprocessing, ablation studies, and comparisons with state-of-the-art methods. A subjective evaluation of the restored image quality is provided, followed by an objective assessment using the peak signal-to-noise ratio (PSNR) and structural similarity index measure (SSIM) [31,32,33] as comparative image quality metrics. PSNR and SSIM primarily quantify differences in luminance, color, and structural features between the compared images. Higher values for both metrics indicate superior image restoration performance.

### 4.1. Experimental Environment Establishment

Software configuration: The experimental code was developed in Python (3.9.20) using the PyCharm (Community Edition 2023.3.2) integrated development environment. The versions of the key libraries employed are as follows: NumPy 1.26.4, Matplotlib 3.9.3, and OpenCV-Python 4.8.1.78.

Hardware setup: Parameter tuning for physical-model-based methods and runtime verification of all approaches were performed exclusively on a CPU processor (Intel i9-13900HX). The deep learning model training utilized a single GPU (NVIDIA GeForce RTX 4060).

Parameter settings: For physical-model-based methods, the brightest pixel selection ratio *p* = 0.3%, the haze removal coefficient *ω* = 0.95, and the lower bound for transmission estimation *t*_0_ = 0.1. The deep-learning-based methods employed the PyTorch (2.5.1) framework with ADAM optimizer. The initial learning rate and batch size were set to 0.00025 and 16, respectively. All deep learning models were trained for 50 epochs.

### 4.2. Image Acquisition and Preprocessing

**Figure 5:**
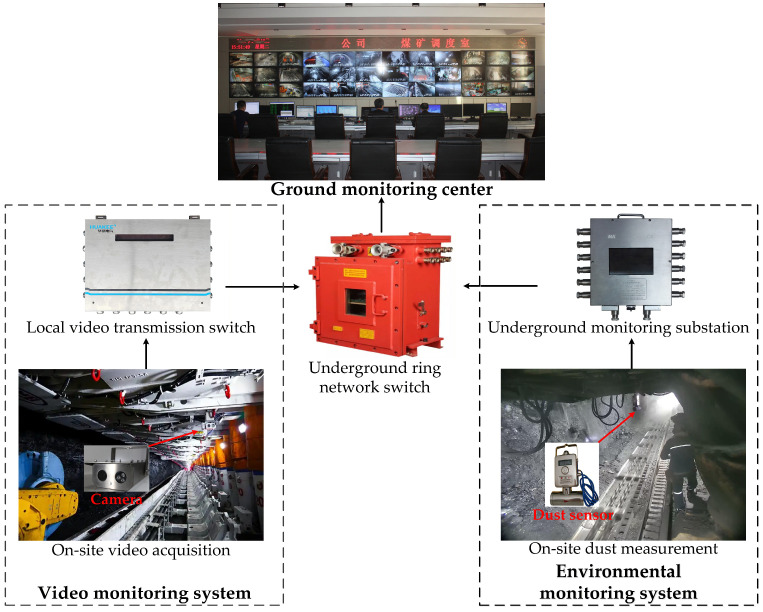
The data acquisition system diagram.

The coal mine hazy image dataset was constructed by the video monitoring system, while dust data were obtained from the environmental monitoring system. The data acquisition system consists of the following components as shown in Figure 5.

The monitoring cameras and dust sensors maintained fixed positions and angles throughout the data collection process. Clear images were acquired during extended production shutdowns for maintenance, while hazy images and dust data were collected in stages during driving or coal mining operations. The coordination between underground dispatch and ground monitoring centers ensured synchronization between image acquisition and dust data collection. A total of 1500 coal mine image pairs were collected, comprising 1000 color image pairs and 500 low-light grayscale image pairs. For the color images, 950 images were randomly selected as the training set, while the remaining 50 images served as the test set. Similarly, for the low-light grayscale images, 450 images were allocated to the training set and 50 images to the test set. Additionally, two public datasets, SOTS-indoor and SOTS-outdoor [34], were incorporated. Both datasets contain 500 images each, with 450 images randomly chosen for training and 50 images for testing in each dataset.

### 4.3. Ablation Studies

#### 4.3.1. Weighted Bright–Dark Channel Fusion

**Figure 6:**
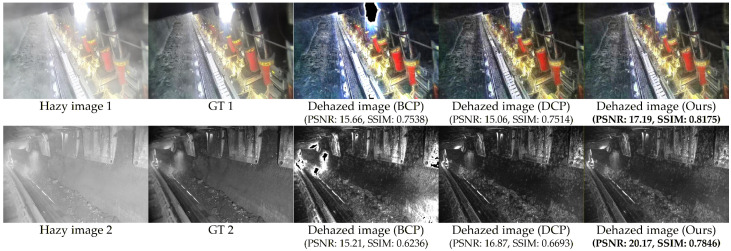
Visual and quantitative comparisons of different estimation methods. The best results are bold.

In the processes of ambient light and transmission estimation, conventional methods typically employ the DCP for transmission estimation. The primary distinction lies in whether ambient light estimation utilizes the BCP or DCP. This study integrates the BCP and DCP through weighted fusion. Comparative analysis with conventional methods was conducted without incorporating weighted guided filter and dust measurement compensation, as illustrated in Figure 6. Hazy image 1 and hazy image 2 represent a color hazy image and a low-light grayscale hazy image, respectively. GT1 and GT2 denote the corresponding clear ground truth images. The dehazed image (BCP) shows results obtained through conventional methods using the BCP for ambient light estimation, which exhibits significant color distortion. Dehazed image (DCP) displays outcomes from methods employing the DCP for ambient light estimation, demonstrating local over-enhancement issues and halo artifacts. Dehazed image (Ours) represents the results obtained through the proposed method. For color images, the optimal metrics achieved are PSNR = 17.19 dB and SSIM = 0.8175. For low-light grayscale images, the optimal metrics are PSNR = 20.17 dB and SSIM = 0.7846. Therefore, compared with conventional methods, the proposed approach demonstrates substantial improvements in both visual image quality and objective evaluation metrics.

#### 4.3.2. Performance Analysis of Weighted Guided Filter and Dust Compensation

**Figure 7:**
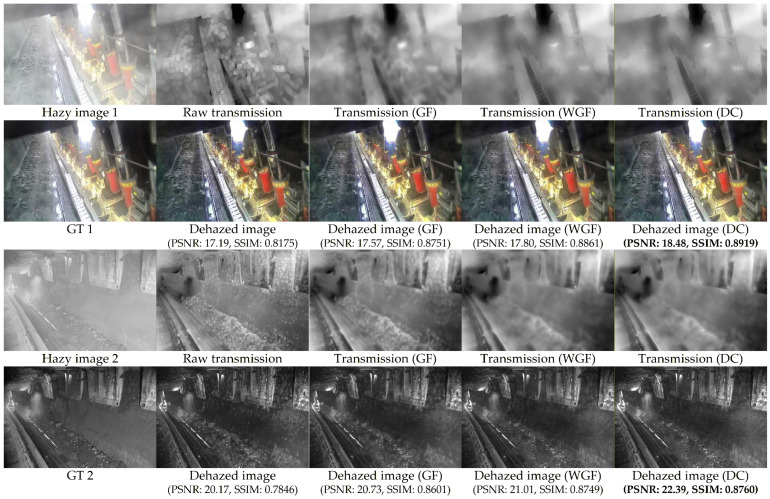
Visual and quantitative comparisons of different methods. The best results are bold.

To further enhance image quality, comparative experiments were conducted by incorporating the proposed weighted guided filter and dust measurement compensation into the weighted bright–dark channel fusion framework. Figure 7 illustrates the experimental results. Raw transmission shows the initial transmission map obtained through weighted bright–dark channel fusion, with the dehazed image representing its corresponding dehazed result. Transmission (GF) displays the transmission map generated by the classical guided filter, while dehazed image (GF) shows the associated dehazed output. Transmission (WGF) illustrates the transmission map produced using our weighted guided filter, and dehazed image (WGF) presents the corresponding dehazed image. Transmission (DC) represents the transmission map obtained by adding dust measurement compensation to the weighted guided filter, with dehazed image (DC) showing the resulting dehazed image. Compared to the original dehazed image, the PSNR increased by 1.29 dB and SSIM improved by 0.0744 for color images. For low-light grayscale images, the PSNR increased by 2.22 dB and SSIM improved by 0.0914. These results demonstrate that incorporating the weighted guided filter and dust measurement compensation significantly enhances key features and edge details in both transmission maps and dehazed images.

#### 4.3.3. Impact of Color Characteristics on Dehazing Performance

**Figure 8:**
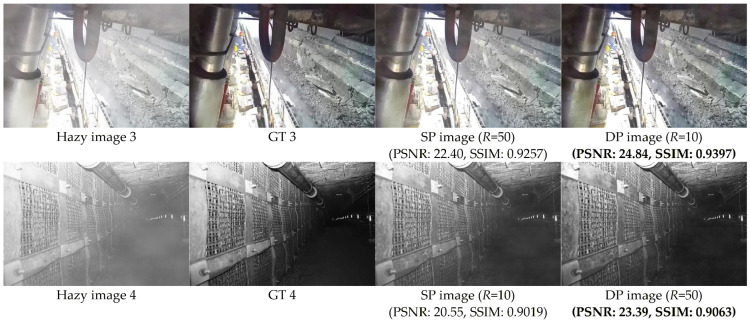
Visual and quantitative comparisons of methods with and without color feature distinction. The best results are bold.

Image color characteristics significantly affect dehazing performance, and hazy image 3 (color image) and hazy image 4 (low-light grayscale image) were selected as the test images, as demonstrated in Figure 8. According to Equation (8), we set different R values for color images (*R* = 10) and low-light grayscale images (*R* = 50), obtaining DP image (*R* = 10) and DP image (*R* = 50), respectively. In contrast, SP image (*R* = 50) represents the dehazed result without color distinction (*R* = 50), while SP image (*R* = 10) shows the result without color distinction (*R* = 10). The comparison reveals that our method achieves superior performance in both visual quality and quantitative metrics, validating the necessity of distinguishing image color characteristics.

#### 4.3.4. Impact of Model Parameters on Dehazing Performance

The model parameters *R* and *α* influence dehazing performance to varying degrees. To determine the optimal parameter combination, we conducted comparative experiments using hazy image 3 and hazy image 4 while keeping other model parameters constant. Table 1 presents the results. For color images, PSNR and SSIM values initially increased and then decreased as *R* varied within the range [1, 80]. The maximum PSNR and SSIM values reached 24.84 dB and 0.9397 respectively when *R* = 10. Similarly, for low-light grayscale images, PSNR and SSIM values followed an increasing-then-decreasing pattern, with maximum values of 23.39 dB and 0.9063 at *R* = 50. These findings indicate that different *R* values should be configured for images with distinct color characteristics to achieve optimal dehazing performance, and adjusting the *R* parameter can significantly enhance dehazing effectiveness.

Table 2 shows that, when *α* takes different values within [0, 1], the PSNR and SSIM for both color images and low-light grayscale images remain relatively stable overall. The metrics reach their peak values at α = 0.8 and α = 0.9, while showing a significant decline when α = 1. Comprehensive analysis indicates that α = 0.8 yields the best overall performance across all metrics. This finding helps determine the reasonable value range for *α*, and the parameter *α* is suitable for fine-tuning dehazing performance.

### 4.4. Comparison with State-of-the-Art Methods

Comparative experiments were conducted using four physics-based methods (DCP [18], TCSF [29], TSDM [30], and our method) and four deep-learning-based methods (AOD-Net [7], PSD [8], RIDCP [9], and MaIR [10]). The experiments utilized a self-created coal mine hazy image dataset along with public SOTS-indoor and SOTS-outdoor datasets. Both subjective visual assessments and objective quantitative metrics with PSNR and SSIM are provided to evaluate the results.

#### 4.4.1. Coal Mine Hazy Image Dataset

Under the influence of dust haze, uneven light distribution, and dark backgrounds, it is essential to restore the features of key targets such as personnel, coal-rock structures, shearers, roadheader, scraper conveyors, hydraulic supports, and other mechanical equipment.

**Figure 9:**
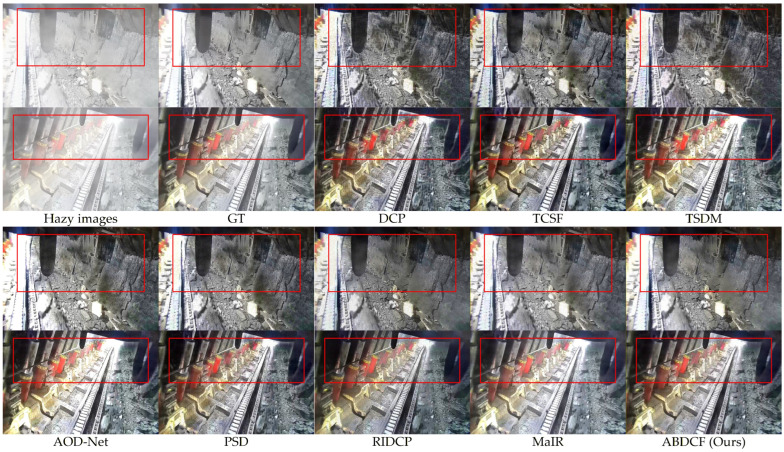
Visual comparisons of different methods for color images.

As shown in Figure 9, for color images under sufficient lighting, the DCP [18] produces color distortion and noise in bright areas. Dark regions show excessive enhancement. Halo artifacts appear on top and side walls. The overall image appears too dark. TCSF [29] reduces halo artifacts but maintains noise in bright areas. Dust remains at the top. TSDM [30] causes excessive enhancement in dark regions. Halo artifacts persist on top and side walls. AOD-Net [7] reduces lighting noise but over-enhances illumination. This causes detail loss in the bottom scraper conveyor. PSD [8] shows excessive enhancement in dark areas with noise in bright regions. RIDCP [9] and MaIR [10] perform similarly, they reduce lighting noise but leave local dust residues.

**Figure 10:**
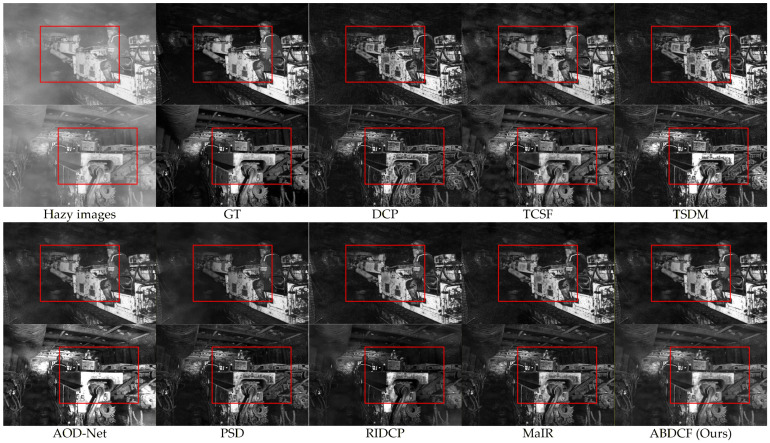
Visual comparisons of different methods for low-light grayscale images.

As shown in Figure 10, for low-light grayscale images under insufficient lighting, the DCP [18] causes excessive enhancement in personnel and equipment areas. Obvious halo artifacts appear. TCSF [29] reduces halo artifacts and excessive enhancement. Local dust residues remain. TSDM [30] achieves good dehazing but shows excessive enhancement and halo artifacts. AOD-Net [7] reduces halo artifacts but significantly increases local exposure. Dark areas retain dust residues. PSD [8] demonstrates excessive enhancement in dark regions with dust residues. RIDCP [9] and MaIR [10] perform similarly with good dehazing effects. However, excessive enhancement occurs in local equipment areas.

In contrast, the proposed method achieves notable dehazing performance while preserving key image features and maintaining a natural appearance, with high color fidelity. According to the quantitative metrics in Table 3, for color images, the proposed method achieves improvements of 1.67 dB in PSNR and 0.0128 in SSIM compared to the second-best MaIR [10]. For low-light grayscale images, the proposed method demonstrates increases of 1.23 dB in PSNR and 0.0164 in SSIM relative to MaIR [10]. Therefore, the proposed method demonstrates superior image restoration performance.

#### 4.4.2. SOTS-Indoor and SOTS-Outdoor Datasets

The SOTS-indoor and SOTS-outdoor datasets serve as established evaluation benchmarks for image dehazing research. These datasets enable objective assessment of algorithm generalization and restoration accuracy. Comparative experiments were performed using both datasets to validate the adaptability of the proposed method. The dust measurement compensation was not employed in Section 4.4.2.

**Figure 11:**
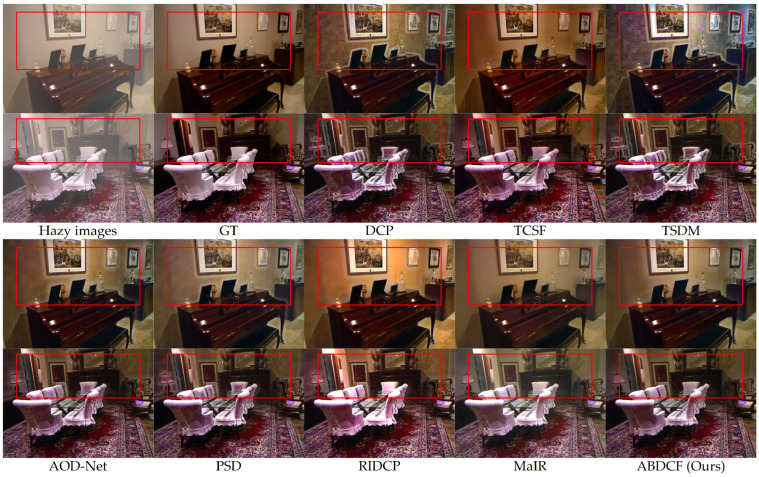
Visual comparisons of different methods for SOTS-indoor dataset.

As shown in Figure 11, for indoor hazy images, the DCP [18] exhibits color distortion in background regions. Dark areas show excessive enhancement. Halo artifacts appear at edges of multiple objects, including tables and chairs. The overall image appears dark. TCSF [29] reduces halo artifacts but causes excessive enhancement in local areas. TSDM [30] demonstrates obvious color distortion in background regions. Dark regions suffer from excessive enhancement. Halo artifacts appear at multiple object edges. AOD-Net [7] and PSD [8] perform similarly. They reduce halo artifacts but cause excessive enhancement in background and chair areas. RIDCP [9] shows color saturation distortion. The overall image appears bright. MaIR [10] restores true features of multiple objects but leaves local dust residues.

**Figure 12:**
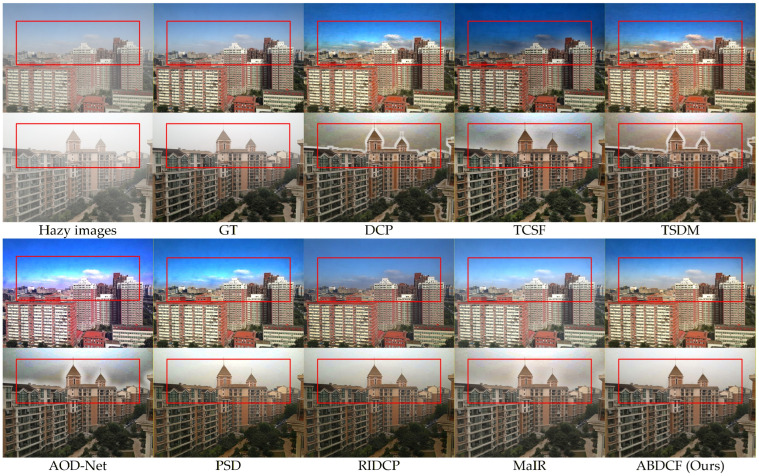
Visual comparisons of different methods for SOTS-outdoor dataset.

As shown in Figure 12, outdoor hazy images show greater sensitivity to lighting variations than indoor hazy images. The DCP [18] shows obvious color distortion in sky and building top areas. Halo artifacts appear at white cloud and building edges. TCSF [29] reduces halo artifacts but causes significant color distortion in sky regions. TSDM [30] causes excessive enhancement in sky and building top areas. Building edges display halo artifacts. AOD-Net [7] exhibits some color distortion in sky and building top areas. Building edges show halo artifacts. PSD [8] demonstrates color distortion in sky areas. RIDCP [9] poorly restores features in sky and local building areas. The overall image appears dark. MaIR [10] causes excessive enhancement in sky and building top areas with local dust residues.

In contrast, the proposed method effectively removes dust haze while fully restoring the key features of multiple targets in the image. The restored images exhibit high contrast and maintain strong consistency with real-world scenes. According to the quantitative metrics in Table 4, for color images, the proposed method outperforms MaIR [10] with PSNR improvement of 1.13 dB and SSIM increase of 0.0149. In low-light grayscale image processing, the method achieves a result 1.49 dB higher in PSNR and 0.0233 greater in SSIM compared to MaIR [10]. Therefore, the proposed method demonstrates excellent image restoration results.

#### 4.4.3. Runtime Comparison and Analysis

The computational efficiency of a model is critical for practical applications, and it is evaluated by testing 10 color images with 620 × 460 resolution on a CPU processor (Intel i9-13900HX). Average runtime served as the evaluation metric. We compared our method, ABDCF, with other state-of-the-art methods in terms of trainable parameter counts and runtime differences. As shown in Table 5, physical-model-based methods contain no trainable parameters, so parameter counts were not reported. These methods demonstrate clear advantages under hardware constraints without GPU support due to low computational complexity. The DCP [18] achieves the shortest runtime at 0.030 s. Our method shows slightly higher runtime at 0.058 s. Deep-learning-based methods exhibit generally longer runtimes. Runtime shows significant positive correlation with parameter counts. Among deep-learning-based methods, AOD-Net [7] exhibits relatively small parameter count and runtime (0.002 M and 0.576 s respectively); RIDCP [9] features the largest model with peak parameter count and runtime (29.67 M and 25.62 s respectively). Our method (ABDCF) maintains extremely low runtime (0.058 s) while providing superior dehazing performance. This effective balance between dehazing quality and computational efficiency demonstrates significant practical value, which facilitates rapid deployment on lightweight terminal devices.

## 5. Conclusions

This study presented a coal mine image dehazing method based on multi-sensor complementarity and adaptive bright–dark channel fusion. It elaborates on key components including image color feature recognition, multi-sensor complementary validation, adaptive bright–dark channel fusion, image edge feature enhancement, and multi-scenario experimental validation. Experimental results demonstrate that, compared to other state-of-the-art methods, the proposed method exhibits superior performance in dehazing effectiveness, stability and efficiency. The main contributions and findings are summarized as follows:

### 5.1. Modeling and Mathematical Contributions

- Impact of color features in atmospheric scattering models: Image color features significantly influence dehazing outcomes. Certain dehazing methods effective for color images perform poorly when processing low-light grayscale images, where the global color mean can serve as an effective discriminative tool.
- Multi-sensor fusion strategy for complex scenarios: Under complex environmental conditions, relying solely on single-sensor image acquisition and signal processing often fails to achieve ideal dehazing results. Employing multi-sensor fusion technology provides a more reliable solution.
- Adaptive adjustment mechanism for parameters: In the estimation of ambient light and transmission, traditional methods frequently depend on empirical parameter settings, which introduce a degree of randomness. Implementing adaptive parameter adjustment based on the image’s intrinsic features represents an effective technical improvement.

### 5.2. Limitations and Future Works

- Image distortion in dense haze scenarios: Existing dehazing methods still exhibit image distortion when processing scenes with high-concentration dust haze, indicating a need for further optimization of related techniques.
- Strong light interference suppression: Beyond dust haze factors, strong light irradiation significantly impacts image restoration quality. Future work could consider incorporating a strong light suppression module to improve system robustness.
- Limited datasets: The underground coal mine environment exhibits complex and variable characteristics. It is difficult to obtain enough real hazy–clear image pairs within limited time periods. Current image datasets do not comprehensively represent all features of underground coal mine environments, and the generalization capability of the proposed method requires further investigation.

## Figures and Tables

**Table 1:** Quantitative comparison of different *R* for dehazing performance. The best results are bold.

*R*	Color Images		Low-Light Grayscale Images	
	PSNR (dB)	SSIM	PSNR (dB)	SSIM
1	19.66	0.9116	15.77	0.7777
10	**24.84**	**0.9397**	20.55	0.9019
20	24.58	0.9387	21.14	0.9033
30	23.81	0.9351	21.89	0.9048
40	23.05	0.9305	22.77	0.9057
50	22.40	0.9257	**23.39**	**0.9063**
60	21.86	0.9209	22.53	0.9000
70	21.42	0.9164	21.70	0.8938
80	21.05	0.9122	20.85	0.8878

**Table 2:** Quantitative comparison of different *α* for dehazing performance.

α	Color Images		Low-Light Grayscale Images	
	PSNR (dB)	SSIM	PSNR (dB)	SSIM
0	24.62	0.9353	23.38	0.9059
0.1	24.68	0.9363	23.36	0.9059
0.2	24.73	0.9373	23.33	0.9061
0.3	24.78	0.9382	23.31	0.9062
0.4	24.81	0.9389	23.28	0.9063
0.5	24.82	0.9393	23.25	0.9065
0.6	24.83	0.9395	23.22	0.9066
0.7	24.84	0.9396	23.22	0.9065
0.8	24.84	0.9397	23.39	0.9063
0.9	24.84	0.9399	23.45	0.9053
1	19.16	0.8757	22.31	0.8851

**Table 3:** Quantitative comparison of different methods for coal mine hazy image dataset. The best results are bold, with the second-best result underlined.

Methods	Color Images		Low-Light Grayscale Images	
	PSNR (dB)	SSIM	PSNR (dB)	SSIM
DCP [18]	17.46	0.8037	17.21	0.7848
TCSF [29]	21.35	0.8370	20.83	0.8214
TSDM [30]	20.43	0.8219	19.36	0.8162
AOD-Net [7]	21.44	0.8472	20.86	0.8314
PSD [8]	20.29	0.8404	19.52	0.8265
RIDCP [9]	21.76	0.8531	20.26	0.8357
MaIR [10]	22.61	0.8764	22.18	0.8592
ABDCF (Ours)	**24.28**	**0.8892**	**23.41**	**0.8756**

**Table 4:** Quantitative comparison of different methods for SOTS-indoor and SOTS-outdoor datasets. The best results are bold, with the second-best result underlined.

Methods	SOTS-Indoor		SOTS-Outdoor	
	PSNR (dB)	SSIM	PSNR (dB)	SSIM
DCP [18]	18.34	0.8279	17.53	0.8048
TCSF [29]	23.81	0.8534	22.65	0.8457
TSDM [30]	21.64	0.8412	20.40	0.8310
AOD-Net [7]	24.27	0.8578	23.19	0.8492
PSD [8]	24.58	0.8612	23.42	0.8515
RIDCP [9]	25.27	0.8769	24.76	0.8561
MaIR [10]	26.73	0.8874	25.46	0.8658
ABDCF (Ours)	**27.86**	**0.9023**	**26.95**	**0.8891**

**Table 5:** Runtime comparisons of different methods. The best results are bold, with the second-best result underlined.

Methods	Params (M)	Runtime (s)
DCP [18]	-	**0.030**
TCSF [29]	-	0.093
TSDM [30]	-	0.119
AOD-Net [7]	0.002	0.576
PSD [8]	6.20	4.87
RIDCP [9]	29.67	25.62
MaIR [10]	3.40	3.25
ABDCF (Ours)	-	0.058

## Data Availability

Data will be made available from the corresponding author on reasonable request.

## Abbreviations

The following abbreviations are used in this manuscript:

DCP	Dark Channel Prior
BCP	Bright Channel Prior
PSNR	Peak Signal-to-Noise Ratio
SSIM	Structural Similarity Index Measurement
